# Seropositivity Status in Patients with Suspected Measles: Are We Going Back to The Beginning with Measles?

**DOI:** 10.5152/eurasianjmed.2026.251181

**Published:** 2026-03-31

**Authors:** Çiğdem Eda Balkan Bozlak, Mahmut Uçar, Özgür Çelebi, Ahmet Yılmaz

**Affiliations:** 1 Department of Microbiology, Kafkas University Faculty of Medicine, Kars, Türkiye; 2 Department of Microbiology, Atatürk University Faculty of Medicine, Yakutiye Municipality, Erzurum, Türkiye; 3 Department of Medical Laboratory Techniques, Atatürk University Vocational School of Health, Erzurum, Türkiye

**Keywords:** Measles, measles IgG, measles IgM, measles vaccine

## Abstract

**Background:**

Measles is a highly contagious viral infection that can cause serious health problems. While measles can be eradicated through vaccination in our country and worldwide, in recent years, vaccine refusal, difficulties in accessing vaccines during the coronavirus disease 2019 (COVID-19) pandemic, and insufficient immunity in young adults have brought the “measles virus” back into the spotlight. This study aimed to compare ELISA test results for measles IgM and IgG in 2024 samples from patients at our hospital suspected of having measles, with respect to age, gender, and season.

**Methods:**

In serum samples taken from patients with suspected measles who came to Kars Kafkas University Faculty of Medicine Medical Microbiology Laboratory, Measles IgG, measles IgM tests (Anti-Measles Virus IgM, Anti-Measles Virus IgG) were studied with kits of the same device in the Enzyme-Linked Immunosorbent Assay (ELISA) method, and the results were evaluated in line with the manufacturer’s recommendations.

**Results:**

In our study, 30 (63.8%) of 47 patients tested were seropositive for measles IgG antibodies. Measles IgM antibodies were detected in 2 (4.3%) of the 47 patients tested. One patient had intermediate values for both measles IgG and IgM.

**Conclusion:**

In our study, no significant relationships were found between age, gender, and season and the Measles IgG and IgM tests. In our study, 34% of the patients in Kars province with measles antibody responses were found to be susceptible to measles infection.

**Main Points**

- In our study, the rate of the measles susceptible population is as high as 34%.
- The COVID-19 pandemic has manifested itself as a resurgence in some infectious diseases, such as measles.
- It is clear that society should be informed that it is possible to protect against measles through vaccination.

## Introduction

Measles is a disease caused by the Rubeola virus, a ribonucleic acid (RNA) virus, which is commonly seen in childhood but has also been encountered in young adults in recent years. It is characterized by fever and maculopapular rash. The measles virus has no known reservoir other than humans, and humans are its primary and only host. It can spread easily from an infected person through droplets (breathing, coughing, sneezing, etc.).^1^

The disease can cause anything from mild symptoms to fatal complications. It is often characterized by high fever, a runny nose, and a rash that spreads throughout the body. The virus can remain active and infectious in the air or on contaminated surfaces for up to 2 hours. Therefore, a person infected with measles can infect 9 out of 10 unvaccinated individuals with whom they have close contact. The infectious period for an infected person typically begins 4 days before the rash appears and continues for 4 days after it appears.^2^^,^^3^ Additionally, due to the congenital transmission of the disease, it is known to cause low birth weight, premature birth, and miscarriage in pregnant women.^4^

Severe complications of the disease are more common in children under 5 and young adults. Measles is widespread in children with weakened immune systems and malnutrition. It is known that vaccine refusals contribute to increased disease.^5^^,^^6^

Before the measles vaccine came into use in 1963, it is estimated that measles caused 2.6 million deaths worldwide each year.^7^^,^^8^When the measles vaccine is administered as a 3-dose regimen for Measles, Mumps, and Rubella (MMR), 96-100% seroconversion can be achieved. According to the plan developed by the World Health Organisation (WHO) in 2024, the disease is planned to be largely eradicated by 2028.^9^

However, due to global vaccination efforts falling short of targeted levels during the coronavirus disease 2019 (COVID-19) pandemic, children are at serious risk from a vaccine-preventable disease like measles.^10^ Therefore, we can say that the rate of measles cases has increased in many developing and underdeveloped societies in the post-pandemic period.^11^ Among young adults, symptoms and sometimes complications of the disease are observed in individuals who were not vaccinated against measles on time or whose immune response did not develop sufficiently. The high-risk group for the disease can be defined as those who have never been vaccinated or, even if vaccinated, have not yet developed immunity.^12^

The measles vaccine was introduced in Turkey in the 1980s and was administered as a single dose in the ninth month for many years. The transition to a second dose of the vaccine began in 1998 with its administration to first-grade primary school students. By 2010, the number of reported cases had dropped to 7. However, due to imported cases from Europe, an outbreak occurred in the winter of 2012-2013, affecting more than 7000 people in our country.^13^

According to a report published by the WHO in October 2024, Turkey reported the highest number of cases in the WHO European Region with 4903 cases between March 2023 and February 2024.^14^

In our study, due to the recent increase in cases at our hospital, we aimed to evaluate the results of the Enzyme-Linked Immunosorbent Assay (ELISA) method for measles IgM and IgG tests in samples sent with suspected measles between 2024 and 2025 from individuals whose serology was not investigated outside of routine prenatal check-ups, considering various factors such as age, gender, and season.

## Material and Methods

Our study was conducted on samples collected from patients in various clinics and services at Kars Kafkas University Hospital between May 29, 2024, and May 29, 2025. In measles cases, IgM antibody positivity can be detected in 70% of patients within the first 2 days and in 90% within 3-5 days of rash onset. Therefore, IgM false negativity within the first 72 hours after rash onset does not exclude the diagnosis, and patients should be followed up. IgM antibodies begin to decline after reaching their peak within 7-10 days, and by the end of 6-8 weeks, they are almost undetectable. IgG levels, on the other hand, peak in the third week and remain in the blood for a prolonged period after infection. The measles virus can also be isolated from clinical specimens, such as nasopharyngeal swabs and urine, up to the first 5 days after the onset of rashes. It can be detected by PCR for up to 7 days (and sometimes after).

In our study, in the serum samples taken from the patients who came to the Medical Microbiology Laboratory of our hospital and wanted to investigate the serology of measles, Measles IgG, measles IgM tests (Anti-Measles Virus IgM, Anti-Measles Virus IgG) were studied in the ALEGRIA (Orgentec Diagnostika GmbH, Mainz, Germany) ELISA device. The results were evaluated according to the manufacturer’s recommendations. After a 1500 RPM refrigerated centrifuge separated the serum samples from the blood samples in tubes with yellow caps for 15 minutes, the samples were loaded into the Allegria device, and the positivity limit was evaluated according to the instructions.

Negative values for Measles (Measles) IgM were evaluated as <20 U/mL, 20-25 U/mL as the cut-off value, and >25 U/mL as positive. Negative values for Measles (Measles) IgG were evaluated as <200 U/mL, 200-250 U/mL as the cut-off value, and positive values above 250 U/mL.

### Statistical Analysis

The data analysis was conducted using SPSS software (IBM SPSS version 24.0 (IBM, ARMONK, NY, USA)). For continuous variables with non-normal distribution, median (minimum–maximum) values were reported. Categorical variables are presented as numbers (n) and percentages (%). Comparisons of categorical variables were performed using the Chi-square test. A *P*-value < .05 was considered statistically significant.

### Ethical Approval

Approval was obtained from the ethics committee of Kars Kafkas University Hospital and the Scientific Research Board of the Ministry of Health for the study (Ethics Committee date and approval No: 29.05.2024 /435). Written informed consent was obtained from all patients who agreed to participate in the study.

## Results

The mean age of the patients included in our study was 25.8 years, with a range of 1 to 65 years. Based on the data obtained within the scope of the study, descriptive statistics regarding the outpatient clinics visited by the participants, the season of admission, and serological test results were evaluated (Table 1).

The most frequently visited unit by participants was the Emergency Clinic, with 29.8% (n = 14) of the total attending. This distribution indicates a significant concentration of emergency visits in the study sample, while other visits were mainly directed to primary care or specific specialties.

Regarding the seasonal distribution, the majority of participants’ visits occurred in spring (48.9%, n = 23). This suggests an increase in visits to healthcare services, particularly during spring months, and may be related to health problems associated with seasonal transitions.

According to serological test results, 63.8% (n = 30) of individuals had positive measles IgG tests, and 2.1% (n = 1) had suspicious/intermediate results. The proportion of individuals with positive measles IgM tests was 4.3% (n = 2), and 2.1% (n = 1) had suspicious/intermediate results. Measles antibodies were not detected in 34% of patients.

Total numbers and results, broken down by gender and season, are presented in Table 2. Results across age groups were not statistically significant (*P* < .05).

Although a higher rate of measles IgG seropositivity was observed in males, there was no statistically significant relationship between gender and IgG test results as a result of the chi-square test performed according to Table 2 (χ²(2)=2.035, *P* = .361). Additionally, the effect size (Cramér’s V = 0.208) was low. Therefore, the IgG result does not reveal a significant gender difference. According to Table 2, there was no statistically significant relationship between gender and IgM test results as a result of the Chi-square test (χ²(2)=0.816, *P* = .665). Additionally, the effect size (Cramér’s V = 0.132) was low. Therefore, the IgM result does not reveal a significant difference between genders. According to Table 2, there was no statistically significant relationship between the season and the results of the IgM test as a result of the Chi-square test (χ²(6)=6.409, *P* = .379). Additionally, the effect size (Cramér’s V = 0.261) was low. Therefore, the IgM result does not indicate a significant difference between seasons. According to Table 3, there was no statistically significant relationship between the outpatient clinic and IgG test results, as indicated by the Chi-square test (χ² = 14.402, *P* = .569). In addition, although the effect size (Cramér’s V = 0.391) was moderate, the difference was not statistically significant. Therefore, the IgG result does not differ significantly across the outpatient clinics. The point emphasized here is that, when we look at the table, 30 of the 47 patients were positive for IgG antibodies, indicating that 14 people in the group known to be fully vaccinated are susceptible to measles.

According to Table 3, there was no statistically significant relationship between age and IgG test results (χ² = 2.904, *P* = .234). Therefore, the IgG test results of individuals do not show a significant difference by age. According to Table 3, there was no statistically significant difference in IgM test results by age (F = 1.497, *P* = .235). Therefore, the IgM test results of individuals do not show a significant difference by age. On the other hand, when we look at the average age of positive patients, the group has a distribution between the ages of 18 and 29, which corresponds to the middle-aged population that is mainly detected in other countries.^6^

According to Table 4, a statistically significant relationship was found between IgG and IgM test results as a result of the Chi-square test (χ² = 47.218, *P* = .00). Additionally, the effect size (Cramér’s V = 0.709) was substantial. This result indicates a strong and significant correlation between IgG and IgM test results. Accordingly, it was evident that IgG had an undeniable effect on immunity. However, acute measles IgM cases were still observed despite low antibody levels in the group.

As shown in the table, the number of measles cases reported from Turkey in 2019 was unclear (Table 5). Especially during the pandemic period, data on certain diseases could not be obtained regularly worldwide.^14^

## Discussion

Measles, one of the diseases that can be prevented by vaccination, has shown periodic increases in recent years due to factors such as anti-vaccination sentiment, irregular migration flows, low immunity among young adults, and the COVID-19 pandemic, which has reduced access to vaccines and fueled anti-vaccination sentiment.^6^^,^^7^ In our city, although measles cases and outbreaks have been rare over the years, an increase in cases was observed during the spring of last year, prompting us to plan this study.

In our study, we examined 47 patients who presented to Kars Kafkas University Medical Faculty Hospital between 2024 and 2025 and underwent measles serology testing for suspected measles. Due to the low number of patients presenting with measles at our hospital in previous years (around 5 per year), requests were made to external laboratories. Still, with the sudden increase in cases, our laboratory began conducting tests in 2024. According to the serological test results, 63.8% (n = 30) of individuals tested positive for measles IgG, suggesting that a significant proportion of the sample may have been previously exposed to measles or developed an immune response through vaccination. The proportion of individuals with negative IgG was 34.0% (n = 16), while the proportion of individuals classified as “intermediate” was limited to 2.1% (n = 1). This data from our study is particularly noteworthy. Although all patients reported receiving a measles vaccination in childhood, antibodies were detected in only 63%. The total number of individuals with low and high antibody levels, even in such a limited population, provides limited information about the prevalence of individuals susceptible to measles.

When the measles IgM test results were examined, 93.6% (n = 44) of patients were negative, 4.3% (n = 2) were positive, and 2.1% (n = 1) were intermediate. Considering that IgM is a marker of acute infection, this distribution can be interpreted as a high proportion of active infection. However, it seems to be low as a percentage in the sample, given the small sample size. When international studies on measles serology are examined, Ledda et al^16^ reported in their Italian study that seropositivity (86%) was higher and sensitivity was higher at younger ages. Fu et al^17^ in their study conducted in China, found that the general measles seroprevalence level was found to be between the ages of 0-60 (70.6%), and the seroprevalence level was found to be between the ages of 15-29 (58.8%). In a measles seroprevalence study conducted by Comas et al^18^ in Spain, the seroprevalence rate was 97.8%. When the studies on measles serology in Turkey are examined, Gündüzalp reported that the level of seropositivity in individuals aged 18-30 in Şanlıurfa city center was 72.9% in 2023.^19^ Emre et al^20^ reported that the measles seropositivity level was reported as 75.8% and that the measles seropositivity level increased with age in their study involving healthcare workers working in clinics with a high probability of encountering measles patients in Izmir in 2023.^20^ Emek et al^21^ in their studies conducted in family health centers in Manisa reported that the seroprevalence of measles was 82.2%. The parameter used in these studies is the presence of measles IgG. That is, it shows individuals in the population who have antibodies to the disease.

When we compare the measles IgG seropositivity rate in our study with these results, we conclude that it is low in our province, indicating that the region’s residents are more susceptible to measles infection. It is assumed that this situation is caused by the immunization rate in our region being lower than in other parts of the country. Again, since the majority of the individuals studied are students, we can see a mixed reflection of people from various provinces in these results. Studies have reported that the measles virus usually spreads in temperate regions at the end of winter and in spring.^6^ In our research in our province, which has a relatively continental climate, 2 measles IgM-positive cases were observed in the spring. This finding supports the literature. However, no statistically significant difference was found.

In the statistical analyses of our study, although there was no significant difference in IgM and IgG positivity by age, season, or patient distribution, noticeable increases were observed at certain times. Especially when we look at the patient group, the information that the origin of the disease is the country in which the epidemic spreads has been determined in the anamnesis taken from some patients. During this period, patients with suspected measles were admitted to our hospital, albeit in small numbers throughout the year. Again, although these periodic increases, which are not statistically significant, raise concerns in society and health institutions about whether measles cases are returning as before, it is understood that this is not the case, as in our example, and that it spreads rapidly from a single source, especially in public living areas. However, the point that should not be overlooked here is that it is often the primary source of positivity in the age group that we can call young adults, which unfortunately brings to mind that we are a society that is open to large epidemics, where adequate immunity does not occur, especially in this group. For this purpose, the importance of measles eradication studies, especially those targeting developing countries, becomes even more evident. We believe that the need for mass measles screenings should also be brought to the agenda to determine the level of measles immunity nationwide, especially in the migration region of our country, and to address the rapid spread of measles in society.

It was observed that measles cases worldwide increased by 79% last year compared to the previous year. In Turkey, the number of cases increased from 51 in 2021 to 125 in 2022 and then to 4,959 in 2023.^8^ The fact that no deaths have been reported in Turkey from the disease, which causes 2-15% mortality, also suggests that there may be issues with the notification system. Overall, although there has been an increase in the number of measles IgM-positive patients worldwide, we believe it has not yet taken its place on the agenda. Although the WHO has set a target to eradicate measles in the European Region by 2010, outbreaks have persisted in many European countries. By the end of 2015, no region other than the Americas had achieved this elimination target. According to the WHO’s 2019 report, the number of reported measles cases increased from 173 000 in 2017 to 229 000 in 2018.^22^ In Turkey, according to Public Health data, the number of positive patients increased from 422 in 2015, 105 in 2016, 100 in 2017, to 638 in 2018.^23^ Between 2019 and 2025, we cannot reach the number of healthy net positive patients and deaths for our country. According to UNICEF’s first reports for 2025, measles cases across Europe have been higher in the last year than in the previous 25 years. They attributed the way to resolve this situation to the need to control vaccination and determine the level of immunity.^24^ Unfortunately, the COVID-19 pandemic has manifested itself in the form of a resurgence of some infectious diseases that are on the verge of eradication. We believe that the increase in vaccine refusals in our country and around the world in recent years is one factor contributing to this situation. In this limited study conducted among patients who applied to our hospital with suspicion in our city, it is seen that society should be informed that it is possible to protect against many infectious diseases, including measles, through vaccination, and that efforts should be increased in this direction, since the rate of measles susceptible population is 34%.

## Figures and Tables

**Table 1.** Distribution of Patients Who Underwent Measles Serology Testing at Our Hospital in Kars Province Over 1 Year, by Service, Season, and Measles IgG And IgM Serology Percentage Results

	**Department**	**N**	**%**
Polyclinic-Department	Family Medicine	8	17.0
	Paediatrics	8	17.0
	Emergency	14	29.8
	Urology	2	4.3
	Neurology Polyclinic	9	19.1
	Internal Medicine	5	10.6
	Gynaecology and Obstetrics	1	2.1
	Brain Surgery	1	2.1
Season	Spring	23	48.9
	Summer	10	21.3
	Autumn	5	10.6
	Winter	9	19.1
Measles IgG	Positive	30	63.8
	Negative	16	34.0
	Greyzone	1	2.1
Measles IgM	Positive	2	4.3
	Negative	44	93.6
	Greyzone	1	2.1

**Table 2.** Comparison of Measles Serology Results in Patients According to Different Variables

**Features**	**Total (n)**	**Measles IgG Serology Result (n)**			*P* value*	**Measles IgM Serology Result (n)**			*P* value*
		**Positive**	**Negative**	**Greyzone**		**Positive**	**Negative**	**Greyzone**	
**Gender**					.361				.6
Male	14	11	3	0		1	13	0	
Female	33	19	13	1		1	31	1	
**Total**	47	30	14	1		2	44	1	
**Season**					.249				.3
Spring	23	12	11	0		2	21	0	
Summer	10	8	2	0		0	10	0	
Autumn	5	4	1	0		0	5	0	
Winter	8	6	2	1		0	8	1	

*Value.

**Table 3.** Comparison of measles IgG and IgM Seropositivity According to Mean Age.

**Measles IgG**	**N**	**Average age Ort.**	**Chi-Square**	***P* Value**
Positive	30	24.58	2.904	.234
Negative	16	21.59		
Greyzone	1	45.00		
**Measles IgM**				
Positive	2	16.50	1.497	.235
Negative	44	25.02		
Greyzone	1	46.00		

**Table 4.** Comparison of Measles IgG and Measles IgM Serology Results Compared to Each Other

		**Positive**	**Negative**	**Greyzone**	**Chi-Square**	***P* Value**	**Cramer’s V**
Positive	Value	1	29	0	47.218	.00	.709
	Expected Value	1.3	28,1	,6			
Negative	Value	1	15	0			
	Expected Value	.7	15,0	,3			
Greyzone	Value	0	0	1			
	Expected Value	.0	,9	,0			

**Table 5.** Table of Measles Cases in the World and Turkey (2015-2024)

**Year**	**World Measles Cases (WHO)**	**Türkiye Measles Cases (HSGM)**	**Resources**
2015	254928	422	WHO
2016	132490	105	WHO
2017	110000	100	WHO
2018	229000	638	WHO (2019 Report)
2019	869770	Undeclared	WHO (2020 Report)
2021	873000	51	WHO
2022	1220000	125	WHO
2023	2180000	4959	WHO
2024	3050000	4903	UNICEF-WHO Europa (2025)

WHO, World Health Organization; UNICEF, United Nations Children’s Fund; HSGM, Turkish Ministry of Health, General Directorate of Public Health.

## Data Availability

The data that support the findings of this study are available on request from the corresponding author.
